# Non-neutralizing antibodies to SARS-Cov-2-related linear epitopes induce psychotic-like behavior in mice

**DOI:** 10.3389/fnmol.2023.1177961

**Published:** 2023-04-17

**Authors:** Jinming Xu, Hui Wei, Pengsheng You, Jiaping Sui, Jianbo Xiu, Wanwan Zhu, Qi Xu

**Affiliations:** ^1^State Key Laboratory of Medical Molecular Biology, Institute of Basic Medical Sciences, Chinese Academy of Medical Sciences, School of Basic Medicine Peking Union Medical College, Beijing, China; ^2^Neuroscience Center, Chinese Academy of Medical Sciences, Beijing, China

**Keywords:** SARS-CoV-2, spike protein, non-neutralizing antibody, glial cell, synaptic plasticity

## Abstract

**Objective:**

An increasing number of studies have reported that numerous patients with coronavirus disease 2019 (COVID-19) and vaccinated individuals have developed central nervous system (CNS) symptoms, and that most of the antibodies in their sera have no virus-neutralizing ability. We tested the hypothesis that non-neutralizing anti-S1-111 IgG induced by the spike protein of severe acute respiratory syndrome coronavirus 2 (SARS-CoV-2) could negatively affect the CNS.

**Methods:**

After 14-day acclimation, the grouped ApoE-/- mice were immunized four times (day 0, day 7, day 14, day 28) with different spike-protein-derived peptides (coupled with KLH) or KLH via subcutaneous injection. Antibody level, state of glial cells, gene expression, prepulse inhibition, locomotor activity, and spatial working memory were assessed from day 21.

**Results:**

An increased level of anti-S1-111 IgG was measured in their sera and brain homogenate after the immunization. Crucially, anti-S1-111 IgG increased the density of microglia, activated microglia, and astrocytes in the hippocampus, and we observed a psychomotor-like behavioral phenotype with defective sensorimotor gating and impaired spontaneity among S1-111-immunized mice. Transcriptome profiling showed that up-regulated genes in S1-111-immunized mice were mainly associated with synaptic plasticity and mental disorders.

**Discussion:**

Our results show that the non-neutralizing antibody anti-S1-111 IgG induced by the spike protein caused a series of psychotic-like changes in model mice by activating glial cells and modulating synaptic plasticity. Preventing the production of anti-S1-111 IgG (or other non-neutralizing antibodies) may be a potential strategy to reduce CNS manifestations in COVID-19 patients and vaccinated individuals.

## Introduction

The current coronavirus disease 2019 (COVID-19) pandemic is caused by severe acute respiratory syndrome coronavirus 2 (SARS-CoV-2), which is consistently plaguing global public health. As of February 14, 2023, more than 756 million cases had been diagnosed, and approximately 6.8 million lives had been claimed (WHO Coronavirus (COVID-19), n.d.). Although the disease mainly affects the respiratory system, an increasing number of studies are indicating that SARS-CoV-2 could also cause an extensive range of neurological complications, such as altered mental status, headache, dizziness, hyposmia, new-onset psychosis, neurocognitive syndrome, and affective disorders (Fotuhi et al., 2020; Mao et al., 2020; Pezzini and Padovani, 2020; Pleasure et al., 2020; Varatharaj et al., 2020; Ariño et al., 2022). Neurological adverse events such as multiple sclerosis and neuromyelitis optica spectrum disorder were reported following COVID-19 vaccines (Mirmosayyeb et al., 2023). However, the mechanism of those central nervous system (CNS) manifestations is still unclear.

Growing evidence suggests that the function of the blood–brain barrier (BBB) could be partly disrupted by SARS-CoV-2. For instance, the elevated inflammatory immune response and cytokine storm could affect endothelial cells and then lead to increased BBB permeability (Fotuhi et al., 2020; Omidian et al., 2022). Furthermore, increased BBB permeability induced by the SARS-CoV-2 envelope protein was also found *in vitro* (Buzhdygan et al., 2020; Ju et al., 2022). Some diseases (such as sepsis) can also lead to dysfunction of the BBB (Tang et al., 2022). A defective BBB may permit latent pathogenic circulating proteins (e.g., antibodies) to enter the CNS, which could ultimately trigger neurological symptoms (Prüss, 2021).

Previous work has indicated that antibodies against the receptor binding domain (RBD) of the spike protein have a virus-neutralizing ability (Jeyanathan et al., 2020). Nevertheless, the RBD of the spike protein is short of the linear epitope, and most antibodies with high signal and response frequency in the sera of COVID-19 patients and convalescent patients are non-neutralizing antibodies (Li et al., 2021). Non-neutralizing antibodies can exert antibody-dependent cytotoxicity (ADCC), but the antibody-dependent enhancement (ADE) and pro-inflammatory effects caused by non-neutralizing antibodies are concerning effects that cannot be ignored (Liu et al., 2019; Vabret et al., 2020). Moreover, microbes can cause host cross-immune reactions due to certain proteins that are homologous or similar to the host, which is called molecular mimicry, and this phenomenon can lead to the occurance of several autoimmune diseases (Cock and Cheesman, 2019). Anti-SARS-CoV-2 antibodies were detected in the cerebrospinal fluid (CSF) of all patients with COVID-19 who had signs of encephalopathy (Alexopoulos et al., 2020). A potential explanation is that pathogenic non-neutralizing antibodies reach the CNS through the leaky BBB and then cause mental disorders by molecular mimicry, ADE or other mechanisms (Liu et al., 2021; Vojdani et al., 2021; Wang et al., 2021; Ariño et al., 2022).

The present study aimed to evaluate whether a non-neutralizing antibody (anti-S1-111 IgG) against the SARS-CoV-2 spike protein could negatively affect the function of the CNS and cause psychotic-like behavior in BBB-deficient mice.

## Materials and methods

### Selection of peptides for immunization

Based on the linear epitope landscape of the spike protein constructed by Li Y et al. (Li et al., 2021), we filtered the epitopes by response frequency, mean level of the control group, mean level of the patient group (Supplementary Table S1). S1-93, S1-111, S1-113, S2-19, and S2-22 were selected as target peptides (high response frequency, high level in the patient group), whereas S1-11 and S2-7 were selected as control peptides (low response frequency, low level in the patient group). All of the above epitopes only trigger non-neutralizing antibodies.

### Animals

*ApoE*^−/−^ mice are a BBB-deficient strain, and the BBB leakage in *ApoE*^−/−^ mice is correlated with a reduction in pericytes and tight junctions (Xu et al., 2019). Therefore, we used *ApoE*^−/−^ mice as a model of BBB leakage to simulate the state of the BBB in patients with COVID-19.

C57BL/6 J male mice (10 weeks old) of *ApoE*^−/−^ genotypes were purchased from ALF Biotechnology (Jiangsu, China), and the mice were acclimated for two weeks before subsequent experimental procedures. All mice lived in a 12-h light/dark cycle at 23 ± 1°C. All experimental procedures were subject to the Guidelines for the Beijing Administration Office of Laboratory Animal Guidelines for the Care and Use of Laboratory Animals and were approved by the Animal Ethics Committee of Peking Union Medical College.

### Preparation of spike-protein-derived peptides and experimental design

All peptides used in this study were added with a cysteine residue during synthesis (1 mg/mL, Sangon Biotech) so that they could be conjugated with keyhole limpet hemocyanin (KLH) (Thermo Fisher, Waltham, MA, United States). KLH-conjugated peptides were used for the immunization of mice, while non-conjugated peptides were applied to ELISA.

**Figure 1:**
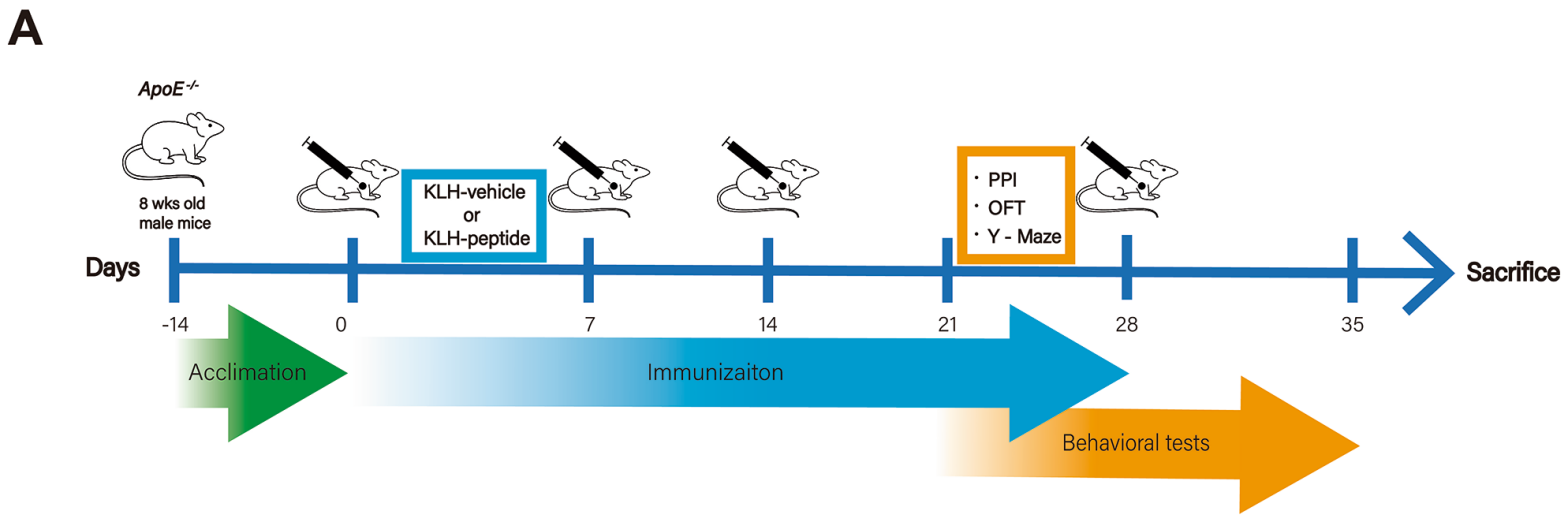
Experimental design. Schematic demonstration of the experimental workflow. *ApoE*^−/−^ (10 weeks) mice were immunized with S protein-derived peptide (coupled with KLH, KLH-peptide) or KLH (KLH-vehicle) only on days 0, 7, 14 and 28. Behavioral tests started on day 23 for the Y-Maze tests, on day 25 for the open field test (OFT), and on day 30 for prepulse inhibition (PPI).

The acclimation, immunization, and behavioral tests were launched as described in the workflow (Figure 1A). Mice received a subcutaneous infusion (day 0, day 7, day 14, day 28) of KLH-conjugated peptide or KLH alone, and the dose (50 μg/mouse, 0.5 mg/mL) was based on that in a previous report (Li et al., 2021). The KLH-peptide and KLH were emulsified in the same volume of Freund’s complete adjuvant (Sigma, MA, United States) for the first immunization, and of Freund’s incomplete adjuvant (Sigma, MA, United States) for the rest of the immunization. The behavioral tests were conducted after the third immunization, and transcriptome profiling and immunochemistry of brain tissue were performed after the sacrifice of the mice (day 35).

On day 35, all mice were sacrificed under anesthesia, and the EDTA anti-coagulant plasma was separated from the blood and stored at −20°C. The brains of mice were split into two hemispheres, one hemisphere was fixed in 4% paraformaldehyde (PFA) (Servicebio, Beijing, China) for immunofluorescence in the regions of interest, and the other hemisphere was used for transcriptome profiling.

### Measurement of anti-S1-111 IgG levels

The presence of anti-S1-111 IgG was examined by ELISA. The S1-111 peptide (10 μg/mL, ECDIPIGAGICAC, Sangon Biotech, China) was covalently ligated on a maleimide-activated 96-well plate, and anti-S1-111 IgG from plasma (1:100) or brain homogenate (1:20) was captured through antigen–antibody reactions. The color signal was visualized by peroxidase-conjugated goat anti-mouse IgG antibody (1:5000, ab6721, Abcam). The optical density at 450 nm (OD_450_) was measured for each sample to evaluate the levels of anti-S1-111 IgG.

### Immunofluorescence

To assess the effects of anti-S1-111 IgG on glial cells, the number of microglia and astrocytes was quantified using immunofluorescence. In brief, the OCT-embedded (Optimum Cutting Temperature Compound, SAKURA, United States) brains were sectioned coronally into 40 μm slices. Then, the sections were incubated with primary antibodies GFAP (1:1000, ab53554, Abcam, United Kingdom), Iba1 (1:1000, ab5076, Abcam, United Kingdom), and CD68 (1:500, 137,001, Biolegend, United States) overnight at 4°C. After washing in 0.01 M PBS three times, the sections were incubated with secondary antibodies donkey anti-rabbit AlexaFluor^®^488 (1:500, A21206, Thermo Fisher, United States), donkey anti-goat AlexaFluor®594 (1:500, A11058, Thermo Fisher, United States), and donkey anti-rat AlexaFluor®594 (1:500, A21209, Thermo Fisher, United States) at 37°C for 1 h. Finally, the sections were mounted and analyzed with a Leica Microscope (TCS SP8 gSTED 3X, Leica, Germany). The mean density of GFAP and the number of microglia were quantified by ImageJ (National Institutes of Health, United States), and microglia (Iba1^+^) colocalized with CD68 were defined as activated microglia.

### Transcriptome analysis and real-time PCR

Total RNA from the cortex and hippocampus was extracted by TRIzol Reagent (Thermo Fisher Scientific, United States) and stored at −80°C. One microgram of total RNA from each sample was used to develop the transcriptome library and conduct sequencing analysis (Novogene, Beijing, China). There were five biological replicates per treatment, and a total of ten libraries were developed. Given that Novogene offers online one-stop analysis tools, we applied DESeq2 to identify the differentially expressed mRNAs (DEmRNAs) between K-S and KLH-immunized mice. GO functional enrichment analysis of DEmRNAs was performed by an online platform provided by Novogene.

cDNA for RT–qPCR was synthesized by the Transcriptor First Strand cDNA Synthesis kit (04897030001, Roche). FastStart Essential DNA Green Master Mix (06924204001, Roche) and a LightCycler 96 Real-Time System (Roche) were used to conduct RT–qPCR with the primers listed in Supplementary Table S2. A fragment sequence of *Gapdh* was introduced as the internal control, and each reaction was performed in triplicate. Gene expression data were calculated by the 2^-ΔΔCt^ method. The primer sequences used in this study are as follows:

*GAPDH*, forward, 5′-GAAGGGCTCATGACCACAGT-3′ and reverse, 5′-GATGCAGGGATGATGTTCTG-3′.

*Rims3*, forward, 5′-AATCCCTCCCAGCCACCTAT-3′ and reverse, 5′-GGCCATACCCATGAAGCACT-3′.

*Lrrk2*, forward, 5′-ACGTGCTTTTTGAGAGAGTTTCC-3′ and reverse, 5′-CATGGCCTCCACCACAAGAT-3′.

*C2cd4c*, forward, 5′-ATCGGAGGAATACGGCTTGG-3′ and reverse, 5′-CAAGAGACCAGGAGTCTGCC-3′.

*Tnr*, forward, 5′-AGGCAAGAGAGGGAGAGAGG-3′, and reverse, 5′-TTGATGCAGACACCCAGGTT-3′.

*Grin2b*, forward, 5′-CCTCCTGTGTGAGAGGAAAGA-3′, and reverse, 5′-TGGTCATTCCCAAAGCGTCC-3′.

*Kcnj2*, forward, 5′-ACTTGCTTCGGCTCATTCTCT-3′, and reverse, 5′-CCAGAGAACTTGTCCTGTTGCT-3′.

*Scn8a*, forward, 5′-GAATGGTCCAAGAATGTGGAGTACA-3′, and reverse, 5′-TCAGTACCCTGAAAGTGCGT-3′.

*Grin2a*, forward, 5′-GGTCAGCTTGAAAACTGGGAAG-3′, and reverse, 5′-TGGTGGCAAAGATGTACCCG-3′.

*Sv2b*, forward, 5′-ACAGGCTCCGTTTAAAGGCTAT-3′, and reverse, 5′-AGGCTTGTGCTGGGAGTAAC-3′.

*Tenm3*, forward, 5′-ACCCAGCTCACAGATACTACC-3′, and reverse, 5′-TCGATTGCCATTCCTTTGGG-3′.

*Shisa6*, forward, 5′-AGCAGACTCCAGGTGATCGT-3′, and reverse, 5′-GAGCGTGAGAAGGAGAGGTC-3′.

*Map1b*, forward, 5′-GACCGTAACCGAAGAGCACC-3′, and reverse, 5′-ATCATCAAACGCACCTCAGTG-3′.

### Behavioral tests

The prepulse inhibition (PPI) level was tested using a sound-controlled box (Xeye Startle Reflex V1.20, Macroambition S&T Development, Beijing, China). The sound control box is sound-proof and light-proof, with a high-quality loudspeaker on the top for generating noise. And a transparent glass box at the bottom where mice can be placed, which is connected to a gravity sensor to detect the startle reflex of the experimental animal. The background noise level was fixed at 65 dB during the whole 25-min experiment, and the test session consisted of four types of PPI trials: background noise alone, acoustic startle alone (120 dB for 40 ms), prepulse alone (70 dB, 75 dB, 80 dB, or 85 dB for 20 ms), and prepulse plus startle (startle after 100 ms of prepulse). The first five minutes was the acclimatization period, then each category of the trial appeared eight times in a pseudorandom order, and the interval between two trials ranged from 10–20 s.

PPI was calculated for each mouse according to the following formula:


$$\begin{array}{l} PPI=100\times [1-\left(\mathrm{startle magnitude for prepulse plus startle}\right)/\\ \;\;\;(\mathrm{startle magnitude for startle stimulus alone})]. \end{array}$$

The open-field test (OFT) was performed in a white polyvinylchloride plastic box (40 cm × 40 cm × 50 cm) in a brightly lit room, and each mouse was placed in the center of the arena and allowed to explore freely for 10 min. The video of exploration was analyzed by ANY-maze (Stoelting, United States) to measure the time in the center (s), total distance traveled (m), and times of entering the center zone. The OFT apparatus was cleaned with 30% ethanol once a mouse finished the test.

Exploratory activity and spatial working memory were evaluated by the Y-maze apparatus (arm length: 40 cm, arm width: 10 cm, height of wall: 15 cm). Every mouse was placed at the center of the Y-maze field and was free to explore the maze for 10 min. Two hind paws completely entering the arm were regarded as an arm entry, and researchers manually counted the number of entries and calculated the alteration. The Y-maze apparatus was cleaned with 30% ethanol once a mouse finished the test.

### Statistical analysis

Statistical analysis was conducted using GraphPad Prism (Version 9.0.0, GraphPad Software, CA, United States). Two-way ANOVA was used to examine the differences in the PPI test between groups, and other experimental data were compared using unpaired Student’s *t* test. Data are shown as the mean ± standard error of the mean (SEM). In all analyses, a *p* value <0.05 was considered statistically significant. ^*^p < 0.05, ^**^p < 0.01, ^***^p < 0.001, ^****^p < 0.0001.

## Results

### KLH-S1-111 immunization induces sensorimotor gating deficiency and anti-S1-111 antibodies in *ApoE* ^−/−^ mice

PPI is a neurological phenomenon that is related to sensorimotor gating, and sensorimotor gating determines whether excess or trivial stimuli are screened as relevant. Deficits in sensorimotor gating are observed in patients with schizophrenia and Alzheimer’s disease (Braff et al., 2001). Therefore, the PPI test was performed as a primary screening test to examine whether immunization could affect the behavior of mice.

**Figure 2:**
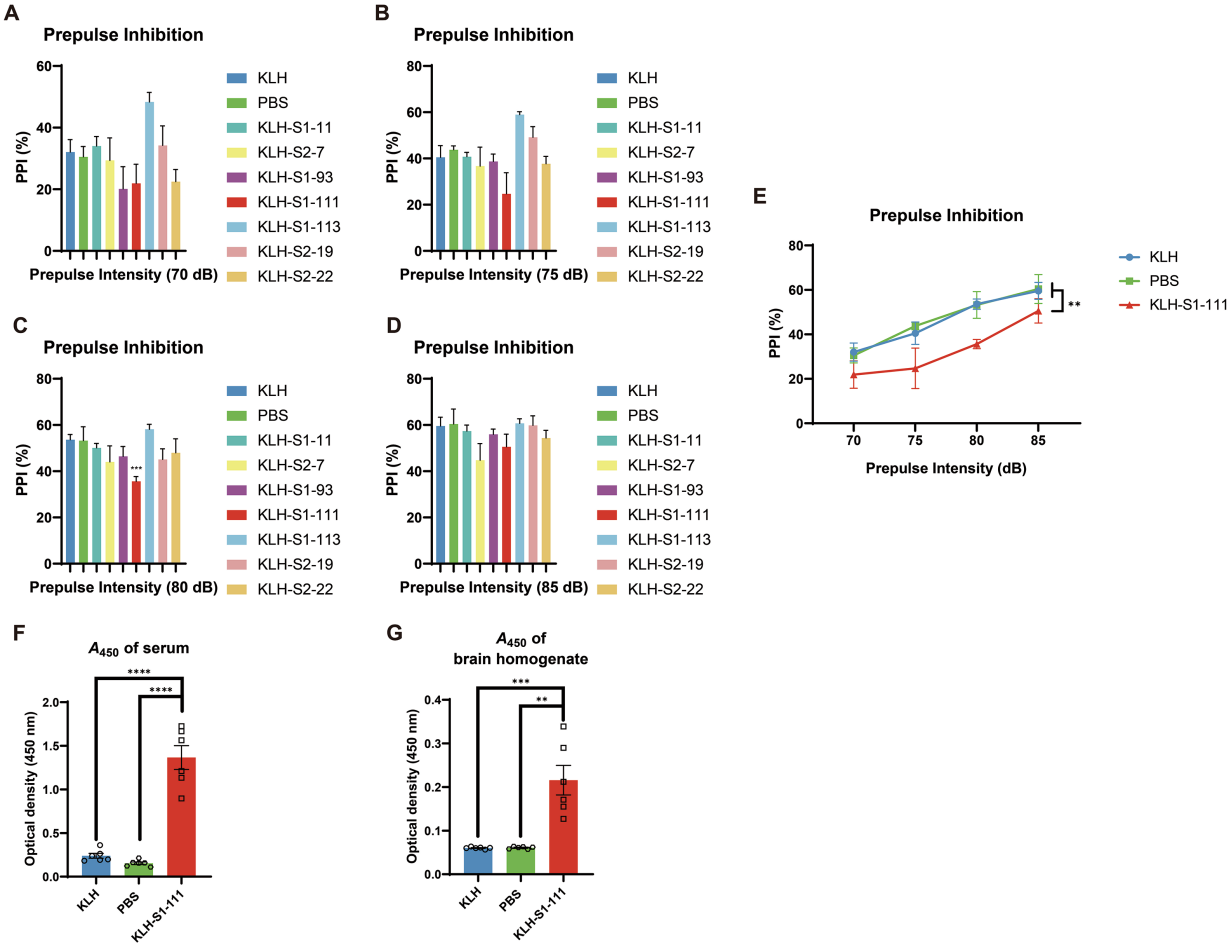
Detection of PPI and antibody levels in immunized *ApoE ^−/−^* mice. **(A–D)** The results of the PPI test at different dBs, and the PPI reduction in K-S mice was significant at 80 dB (unpaired Student’s *t* test, *p* = 0.0004). **(E)** A significant reduction in % PPI (two-way ANOVA, *p* = 0.0011) was found in K-S mice compared with KLH-immunized mice (*N* = 5/group). **(F–G)** Increased anti-S1-111 IgG levels were measured in serum (unpaired Student’s *t* test, *p* < 0.0001) and brain homogenate (unpaired Student’s *t* test, *p* = 0.001) of K-S mice compared with KLH-immunized mice. *N* = 6/group, unpaired Student’s *t* test. **(A–G)**. Data are shown as the mean ± SEM.

As shown in Figure 2E, only the PPI of the KLH-S1-111-immunized (K-S) group exhibited a significant downward trend compared with that of KLH- and PBS- immunized groups (**significant reduction at 80 dB,** Figures 2A-D), which indicated the K-S group developed sensorimotor gating deficiency. Consequently, S1-111 was chosen as the target linear epitope for the subsequent detailed experiment. Serum and brain homogenate were diluted at 1:100 and 1:20, respectively. ELISA was used to detect the level of antibodies in the samples. Compared with the PBS-immunized group and KLH-immunized group, the K-S group had high levels of specific anti-S1-111 IgG in plasma (Figure 2F) and brain homogenate (Figure 2G) after 4 weeks of immunization. This findings suggested that antibodies targeting S1-111 were present not only in the blood circulation system but also in the brain.

### KLH-S1-111 immunization induces glial cell activation in *ApoE* ^−/−^ mice

As a result of the increased levels of anti-S1-111 IgG was measured in K-S mice, we introduced immunofluorescence to determine the activation state of glial cells and evaluate pathologies. In immunofluorescence experiment, Iba-1 was used to mark microglia, GFAP^+^ was used to mark astrocytes, and CD68 was used to mark activated microglia.

**Figure 3:**
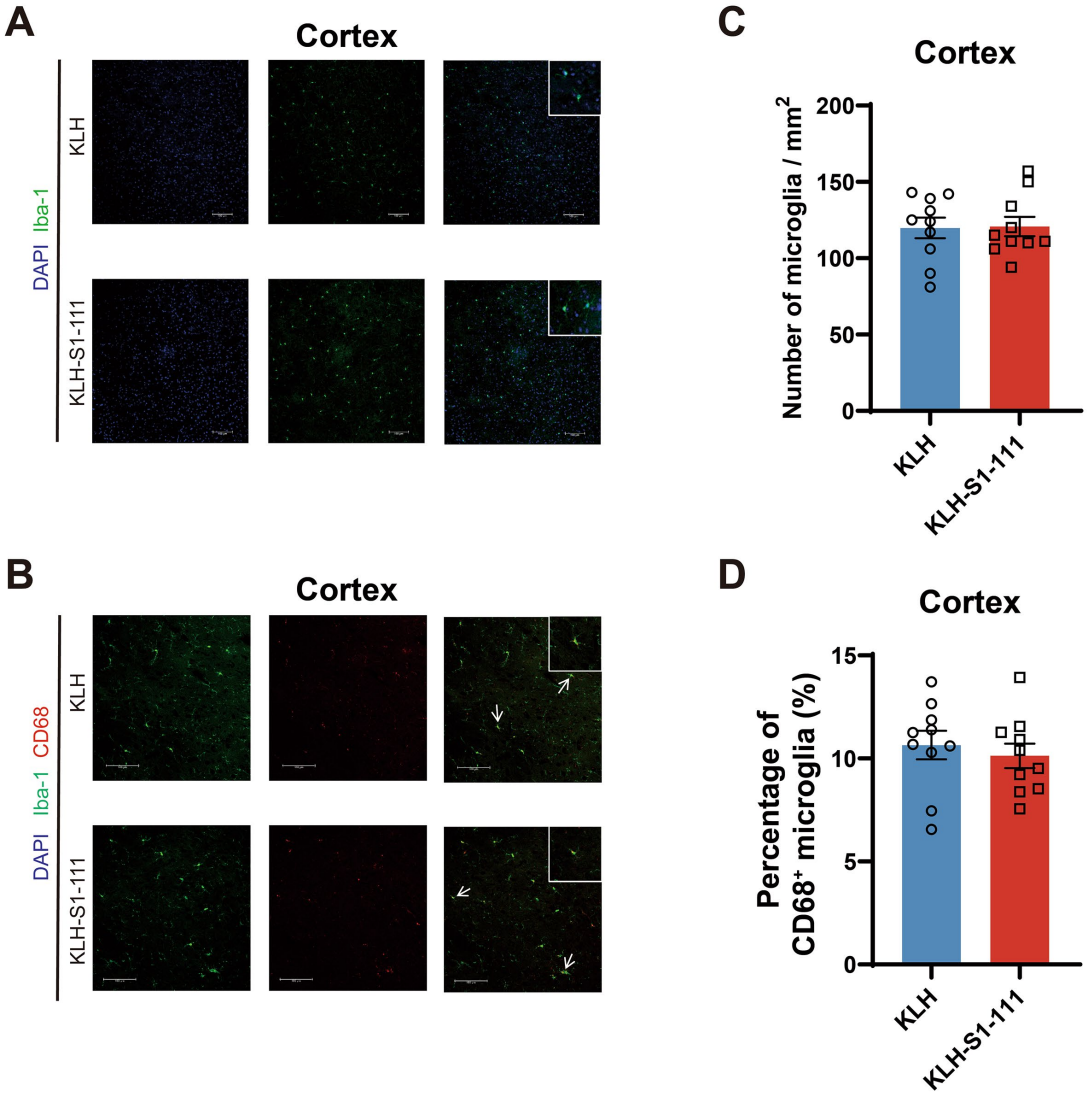
Evaluating of the effect of endogenous anti-S1-111 antibodies on cortical glial cells. Representative images depicting immunofluorescence staining in the cortex **(A)** for Iba1^+^ microglia, and **(B)** for Iba1^+^ and CD68^+^ microglia. Magnification 20× objective field with 100 μm scale bars for A and B; white boxes indicate the enlarged insert of a representative cell with 10 μm scale bars. **(C)** Quantification of Iba1^+^ microglia. **(D)** Quantification of Iba1^+^ and CD68^+^ double-positive area in Iba1^+^ area. **(C,D)**. *N* = 10/group, unpaired Student’s *t* test, data are shown as the mean ± SEM.

In the cortex, no differences were observed in the density of Iba1^+^ microglia (Figures 3A,C) or Iba-1^+^ and CD68^+^ microglia (Figures 3B,D) between the KLH-immunized group and the K-S group.

**Figure 4:**
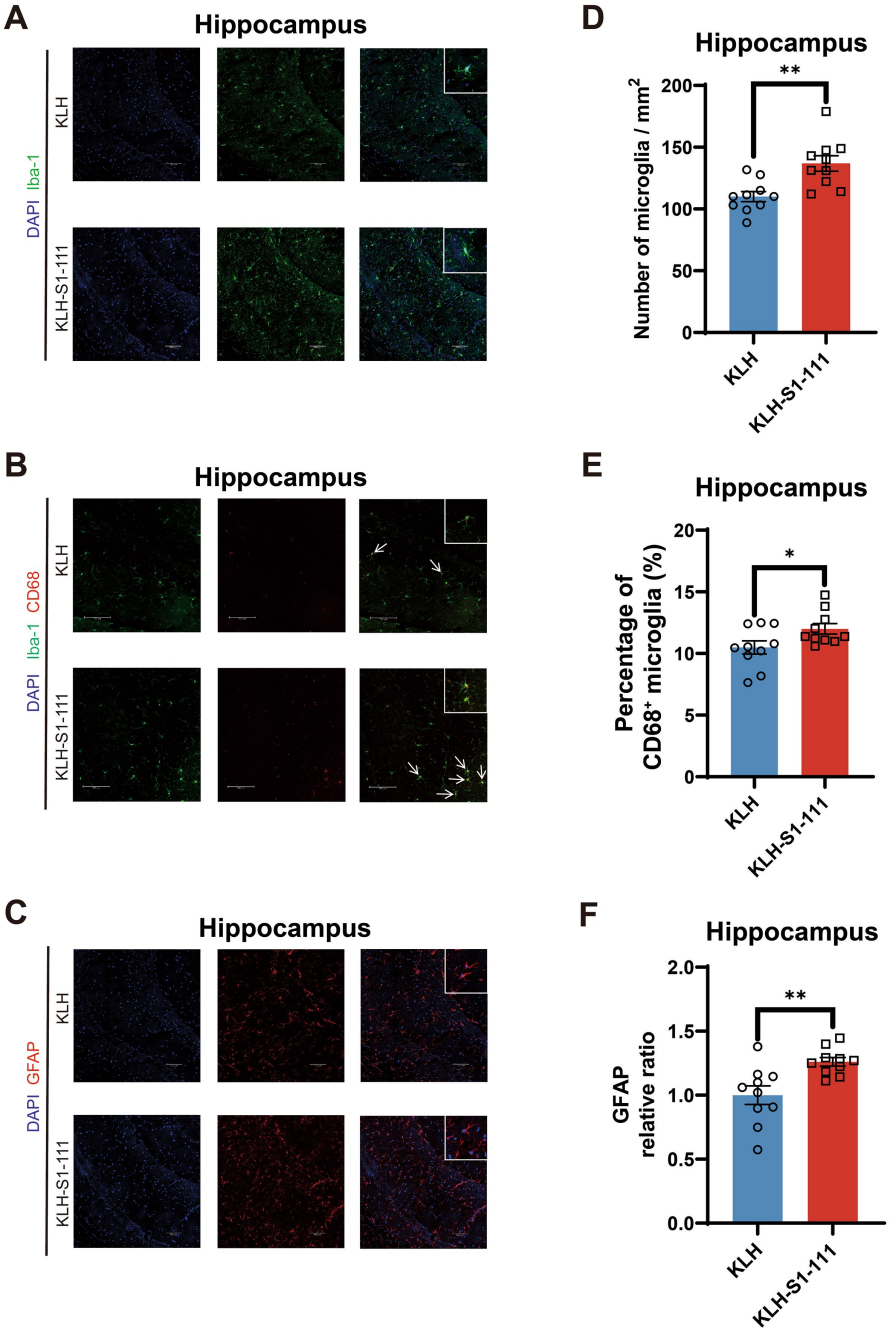
Evaluating of the effect of endogenous anti-S1-111 antibodies on hippocampal glial cells. Representative images depicting immunofluorescence staining in the hippocampus **(A)** for Iba1^+^ microglia, **(B)** for Iba1^+^ and CD68^+^ microglia, and **(C)** for GFAP^+^ astrocytes. Magnification 20× objective field with 100 μm scale bars for A and B, and magnification 40× objective field with 100 μm scale bars for C; white boxes indicate the enlarged insert of a representative cell with 10 μm scale bars. **(D)** Quantification of Iba1+ microglia cells (*p* = 0.002). **(E)** Quantification of Iba-1^+^ and CD68^+^ double-positive area in Iba1^+^ area (*p* = 0.0419). **(F)** Quantification of GFAP^+^ astrocytes (*p* = 0.0041). **(D-F)**. N = 10/group, unpaired Student’s *t* test, data are shown as the mean ± SEM.

In the hippocampus, compared with KLH-immunized mice, K-S mice exhibited an increased density of Iba1^+^ microglia with larger cell bodies (Figures 4A,D), and showed an elevated density of Iba-1^+^ and CD68^+^ microglia (Figures 4B,E). Moreover, an increased density of GFAP^+^ astrocytes was also observed in the hippocampus (Figures 4C,F). These results suggest that glial cells were activated in the hippocampal region but not in the cortical region of K-S mice.

### KLH-S1-111 immunization induces gene expression changes in *ApoE*^−/−^ mice

**Figure 5:**
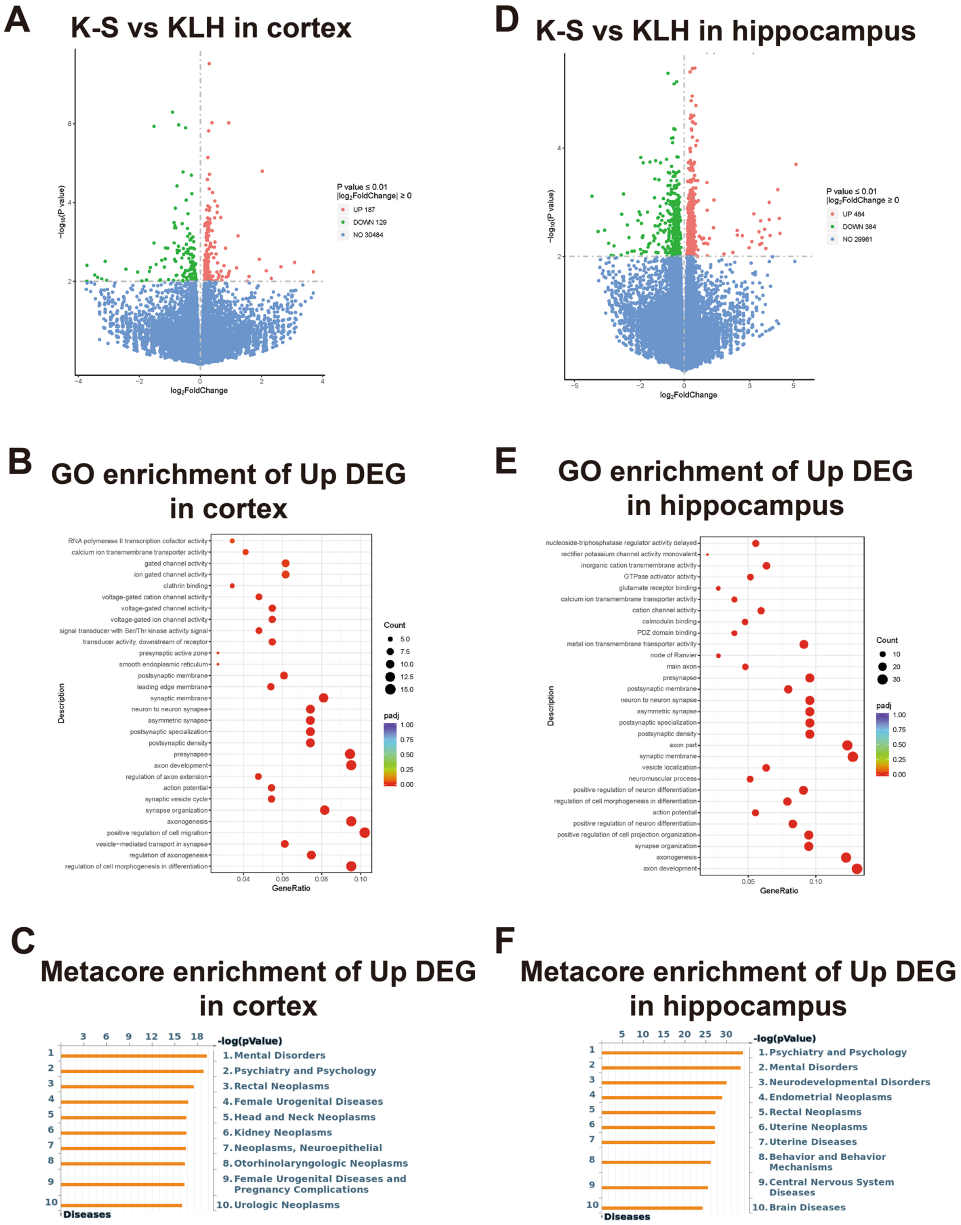
The enriched functional analysis and transcriptional profiles of DEmRNAs. **(A,D)** Volcano plot pictures show log_2_FC values and -log10 (*p* value) of mRNAs in immunized mice. **(B,E)** Bubble plot showing the GO enrichment of up-regulated genes. **(C,F)** Bubble plot showing the Metacore enrichment of up-regulated genes.

From RNAseq data, we identified 54,532 protein-coding genes in the cortex and hippocampus of immunized mice by using *Mus musculus* (GRCm38/mm10) as a reference genome, and their log_2_FC values are presented as volcano plots in Figure 5A and Figure 5D. Corresponding to the immunofluorescence results, the number of DEmRNAs in the hippocampus was significantly higher than that in the cortex, suggesting that the hippocampus had more predominant responses to anti-S1-111 IgG. A heatmap showed the expression profiles of up-regulated neuron-related genes in immunized mice (Supplementary Figures S1A and S1B).

To explore the biological functions of the up-regulated genes in K-S mice, GO analysis and Metacore disease analysis were performed. In the cortex, GO analysis demonstrated that up-regulated genes were mainly enriched in the pathways regulating axonogenesis, axon development, synapse organization (Figure 5B). Metacore disease analysis showed that up-regulated genes were primarily enriched in mental disorders, psychiatry and psychology (Figure 5C). In the hippocampus, GO analysis revealed that up-regulated genes were mostly enriched in pathways regulating axon development, axonogenesis, and synapse organization (Figure 5E). Metacore disease analysis revealed that up-regulated genes were principally enriched in psychiatry and psychology, and mental disorders (Figure 5F).

### KLH-S1-111 immunization induces psychosis-like behaviors in *ApoE* ^−/−^ mice

**Figure 6:**
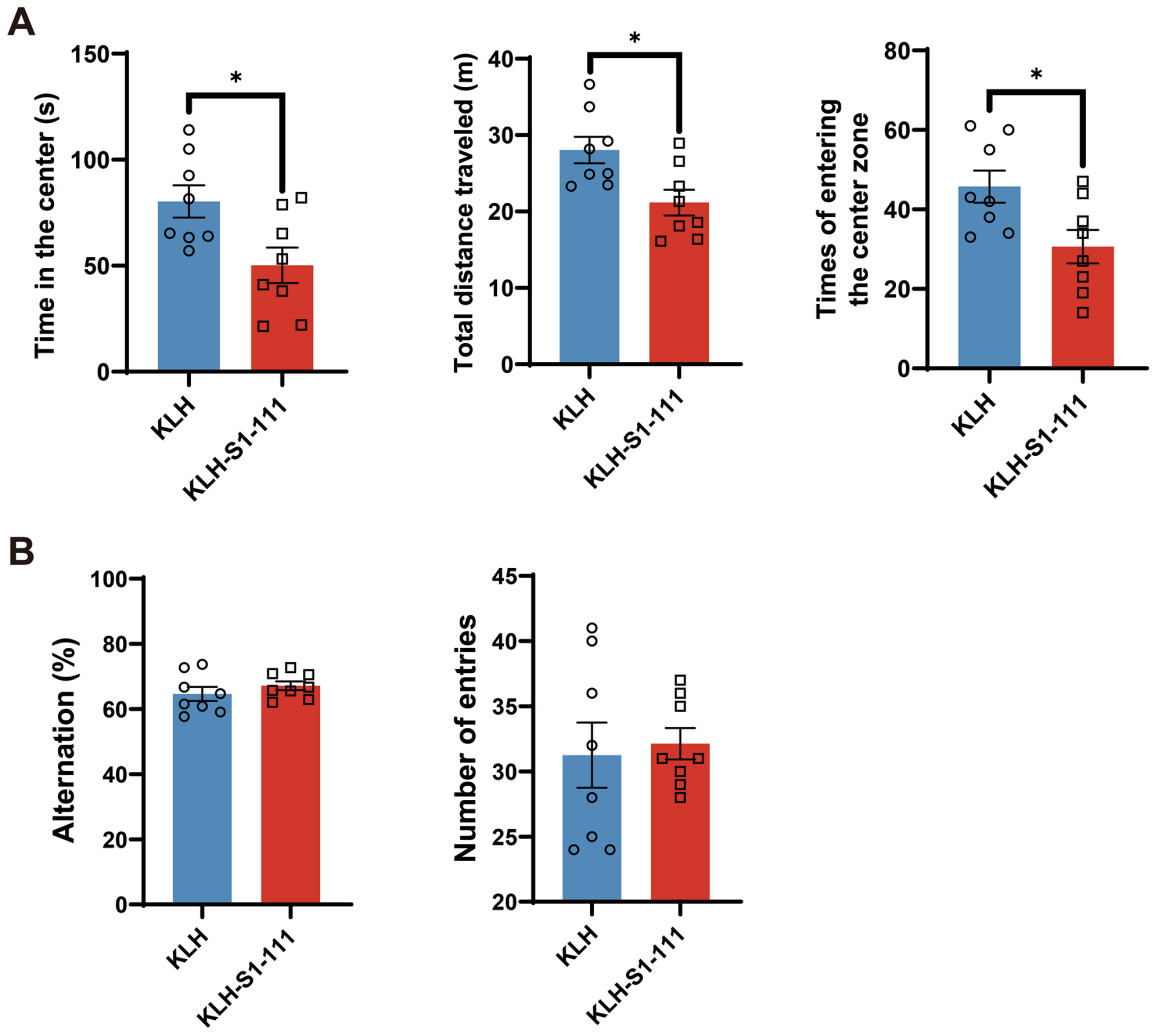
Behavioral effects of endogenous anti-KLH-S1-111 IgG antibodies in *ApoE*^−/−^ mice. **(A)** Open field test (OFT). The time in the center (*p* = 0.0185), total distance travelled (*p* = 0.0131) and number of times entering the center zone (*p* = 0.0207) were significantly decreased in the K-S group, unpaired Student’s *t* test. **(B)** Y maze test. The histogram shows no significant differences in the alternation rate or number of entries between the two immunized groups. **(A,B)**. *N* = 8/group, data are shown as the mean ± SEM.

Based on the results of transcriptome profiling, we conducted a series of behavioral tests to investigate whether K-S mice developed psychosis-like behaviors. Consistent with the results of the screening stage, K-S mice showed a strong reduction in PPI (Supplementary Figures S2A and S2B), implying that sensorimotor gating was impaired in K-S mice. Additionally, the OFT was performed to further evaluate the effects of KLH-S1-111 immunization on locomotor activity. Compared with the KLH-immunized group, the K-S group exhibited significantly reduced time spent in the center, total distance traveled, and number of entries into the center (Figure 6A), suggesting that K-S mice developed anxiety-like and depressive behaviors.

However, there were no significant differences between the KLH-immunized group and the K-S group in the Y-maze test (Figure 6B), indicating that short-term spatial working memory was not impaired.

### Verification of sequencing results by RT–qPCR

**Figure 7:**
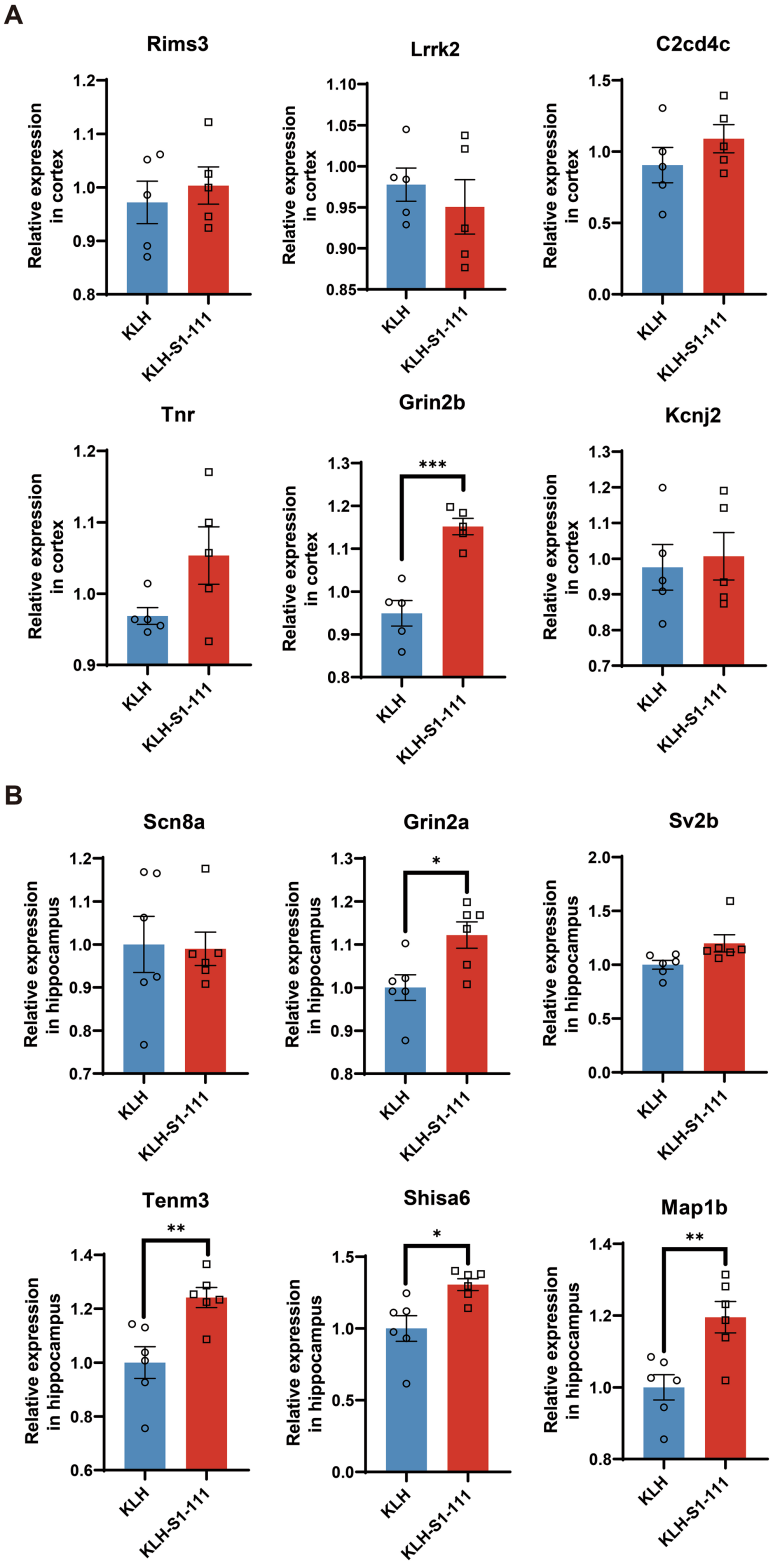
mRNA expression levels of immunized mice measured by RT–qPCR. **(A)** The RT–qPCR results in the cortex, *N* = 5/group, *p* = 0.0004 for *Gin2b*. **(B)** The RT–qPCR results in the hippocampus, *N* = 6/group, *p* = 0.0169 for *Gin2a*, *p* = 0.006 for *Tenm3*, *p* = 0.0112 for *Shisa6*, *p* = 0.0059 for *Map1b*. **(A,B)**. Unpaired Student’s *t* test, data are shown as teh mean ± SEM.

According to the results of transcriptomic sequencing, the expression levels of *Rims3*, *Lrrk2*, *C2cd4c*, *Tnr*, *Grin2b*, and *Kcnj2* were significantly up-regulated in the cortex of the K-S group; the expression levels of *Scn8a*, *Grin2a*, *Sv2b*, *Tenm3*, *Shisa6*, and *Map1b* were significantly up-regulated in the hippocampus of the K-S group. The RT–qPCR results showed that *Grin2b* expression in the cortex (Figure 7A) and *Gin2a*, *Tenm3*, *Shisa6*, and *Map1b* expression in the hippocampus (Figure 7B) were consistent with the results acquired by transcriptomic sequencing.

## Discussion

Numerous studies have reported that neurological manifestations including headache, anorexia, anxiety, dizziness, and loss of smell occur in patients with COVID-19 (Erickson et al., 2021) and vaccinated individuals (al Khames Aga et al., 2021). These adverse events indicate that the CNS could be impaired after being infected with SARS-CoV-2 or vaccinated for the disease. It has been reported that consistent exposure to high levels of cytokines or the direct infection of neurons by SARS-CoV-2 may account for neuropsychiatric symptoms (Fotuhi et al., 2020; Zhang et al., 2021). However, it is unknown whether these symptoms are directly caused by the virus, vaccines, or their products. In this study, we investigated whether anti-S1-111 IgG had the ability to affect normal CNS function in *ApoE^−/−^* mice. Anti-S1-111 IgG was able to activate glial cells, modulate synaptic plasticity, and trigger behavioral abnormalities.

A previous study used a peptide microarray to construct the linear epitope landscape of the spike protein of SARS-CoV-2, and researchers also examined the virus-neutralizing ability of antibodies corresponding to 14 peptides (12 AA per peptide) derived from the spike protein. Furthermore, the titer of anti-S1-111 IgG peaked approximately 30 days after the infection with COVID-19, and the response frequency was 49.7% in patients (Li et al., 2021). Based on the above data, the 6 potential target peptides selected in the screening stage could induce corresponding non-neutralizing antibodies in mice. In our study, anti-S1-111 IgG was detected in both serum and brain homogenate.

Prepulse inhibition (PPI) is a neurological phenomenon that is related to the ability to filter out unnecessary information, and deficits in PPI were observed not only in patients with schizophrenia but also in patients with panic disorder, obsessive–compulsive disorder, and attention deficit disorder (Ong et al., 2005; Kim et al., 2021). Thus, PPI deficit is an indicator of malfunction in a specific brain circuit, and we introduced PPI as a screening method to help select the target peptide in this study. After immunization with several peptides, although other peptide-immunized mice exhibited a tendency toward PPI reduction (such as KLH-S1-93), only K-S mice exhibited a significant reduction in PPI. Therefore, KLH-S1-111 was selected as the target peptide in this study.

Iba-1 is a calcium-binding protein specifically expressed in macrophages and microglia; and CD68 is associated with lysosomes, which is a marker of activated and phagocytic microglia (Fracassi et al., 2022). Moreover, glial fibrillary acidic protein (GFAP) is a marker of activated astrocytes and was applied to evaluate the activation of astrocytes in this study (Jiang et al., 2022). The immunofluorescence results showed that the number of microglia and the number of activated microglia and astrocytes were increased in K-S mice. Although the hippocampus is considered to be related to memory, spatial working memory was not affected by the abnormally increased and activated glial cells according to the results of the Y-maze test. Nevertheless, decreased locomotor activity evaluated by open field test (OFT) indicated that the KLH-S1-111-immunized mice were in a state of depression and anxiety, and the reduction in PPI suggested that sensorimotor gating was deficient. Consequently, the presence of anti-S1-111 IgG certainly influenced the condition of glial cells and behavior in our mouse model. It has been reported that microglia can shape synaptic plasticity and offer trophic support in the mature brain (Guedes et al., 2022). Intriguingly, the up-regulated genes in K-S mice were found to be related to synapse organization and mental disorders according to the transcriptome profiling results. In the cortex, *Grin2b* is involved in the regulation of dendritic spine maintenance and neuron death, and the expression of *Grin2b* was up-regulated in the prefrontal cortex of a schizophrenia rat model (Elfving et al., 2019). In the hippocampus, *Grin2a* plays a crucial role in synaptogenesis and synaptic plasticity (Marwick et al., 2015), *Tenm3* may orchestrate the assembly of topographic circuit assembly in the hippocampus (Berns et al., 2018), *Shisa6* is a plausible candidate gene for Alzheimer’s disease and is associated with post-synaptic transmission (Ramos et al., 2022), and up-regulated *Map1b* may promote axon growth (Wang et al., 2022). Based on the above findings, we suppose that COVID-19-induced immune changes could affect gene expression in the brain, leading to altered neuronal circuits and synaptic plasticity, which could ultimately result in CNS-related disorders.

Infection of SARS-CoV-2 can promote the activation of B cells during the acute phase, then mature B cells will present the viral antigen to CD4^+^ T cells and consequently induce rapid antibody clonal expansion and diversification in patients with COVID-19 (Zhang et al., 2022). In this case, the similarity between specific antigens of SARS-CoV-2 and endogenous protein may cause cross-reaction and trigger an autoimmune reaction. Not all antibodies induced by the SARS-CoV-2 possess the ability of virus-neutralizing, and non-neutralizing antibodies with high titers that persistently exist in the circulatory system may cause multi-system adverse reactions. For instance, autoantibodies induced by SARS-CoV-2 against type I IFNs were detected in 10.2% of life-threatening COVID-19 patients, and this autoimmune reaction is considered to be responsible for the development of severe patients (Bastard et al., 2020). Besides the respiratory system, if the antigen of SARS-CoV-2 is highly homologous with the nervous system tissue, then the cross-reactive antibody can also trigger the corresponding autoimmune response. For example, Epstein–Barr virus (EBV) infection has been linked to multiple sclerosis (Lanz et al., 2022), and *Campylobacter jejuni* is responsible for about a third of Guillain-Barré cases (Finsterer, 2022). According to the BLAST analysis (Supplementary Table S2), S1-111 is homologous to the OCA2 protein, which is hypothesized to be an integral membrane protein involved in small molecule transport, specifically tyrosine (Yang et al., 2019). In addition, OCA2 is a transmembrane protein, which is reported to be expressed in the choroid plexus epithelium, prefrontal cortex and hippocampus (OCA2, n.d.), and is mainly expressed in neuronal cells and glial cells (Single cell type, n.d.). Above all, anti-S1-111 IgG may stimulate the autoimmune response of OCA2 through cross-reaction in the CNS, thereby changing various phenotypes of *ApoE^−/−^* mice.

This study has several important limitations. First, the selection method of peptides in this study was relatively simple, but a previous study indicated that the neurological symptoms caused by COVID-19 are diverse (Zhang et al., 2021). The PPI test used in the selection is commonly regarded as an operational assessment of sensorimotor gating (Takase et al., 2022), which only examines the behavioral change from one dimension. As a result, we might have missed several potentially harmful non-neutralizing antibodies that lead to other symptoms. Therefore, a multidimensional selection strategy that includes as many behavioral tests as possible is needed in future studies. Second, the pathogenesis of anti-S1-111 IgG was not revealed in this study. In our study, we only observed that the level of anti-S1-111 IgG antibody was increased, and glial cells and behavioral results were altered in *ApoE^−/−^* mice. More crucially, the relationship between anti-S1-111 antibody and pathological changes is still unclear. Thus, further studies are required to elucidate the relationship between non neutralizing antibodies and COVID-19-related neuropsychiatric manifestations.

In conclusion, our study showed that the anti-S1-111 IgG non-neutralizing antibody could stably exist in the circulatory system and could reach the CNS when the BBB is dysfunctional, leading to neurological manifestations. People with medical conditions that might lead to leakage of the BBB should take precaution to avoid infection with SARS-CoV-2 and carefully consider vaccination. Importantly, our findings suggest that blocking the production of the non-neutralizing antibodies could be a strategy for preventing COVID-related neuropsychiatric symptoms. Moreover, in the development stage of vaccines, researchers can intentionally reduce the proportion of sequences corresponding to non-neutralizing antibodies to minimize the side effects of vaccines.

## Data availability statement

The datasets presented in this study can be found in online repositories. The names of the repository/repositories and accession number(s) can be found in the article/Supplementary material.

## Ethics Statement

The animal study was reviewed and approved by Animal Ethics Committee of Peking Union Medical College

## Author contributions

JXu performed the main experiments, analyzed the data, and drafted the manuscript. PY and JS conducted the behavioral tests. JXi and WZ conducted the serological tests. QX and HW participated in the experimental design and revised the manuscript. All authors contributed to the article and approved the submitted version.

## Funding

This work was supported by research grants from the National Natural Science Foundation of China (81930104, 32293213, 82071504 and 82101545), the STI2030-Major Projects (2021ZD0203000, 2021ZD0203001, 2022ZD0211700, 2022ZD0211701), the National Key Research and Development Program of China (2020YFA0804502), and CAMS Innovation Fund for Medical Sciences (2021-I2M-1-020).

## Conflict of interest

The authors declare that the research was conducted in the absence of any commercial or financial relationships that could be construed as a potential conflict of interest.

## Publisher’s note

All claims expressed in this article are solely those of the authors and do not necessarily represent those of their affiliated organizations, or those of the publisher, the editors and the reviewers. Any product that may be evaluated in this article, or claim that may be made by its manufacturer, is not guaranteed or endorsed by the publisher.

## Abbreviations

ApoE: apolipoprotein E
BBB: blood–brain barrier
CNS: central nervous system
COVID-19: coronavirus disease 2019
ELISA: enzyme-linked immunosorbent assay
GFAP: glial fibrillary acidic protein
KLH: keyhole Limpet Hemocyanin
LA: locomotor activity
OCT: optimal cutting temperature compound
OFT: open field test
PFA: paraformaldehyde
PPI: prepulse inhibition
RBD: receptor bingding domain
