# Supplementary material for: Non-neutralizing antibodies to SARS-Cov-2-related linear epitopes induce psychotic-like behavior in mice
Source: Front Mol Neurosci. 2023 Apr 17;16:1177961. doi: 10.3389/fnmol.2023.1177961 (PMC10149951; doi:10.3389/fnmol.2023.1177961)
Supplement: Supplementary file 1 [file Data_Sheet_1.docx]

**Supplementary Table 1. Information for S-protein-derived peptide.**

| **Peptide ID** | **Start Position** | **Amino acid sequence** | **End Position** | **Response**  **frequency** | **Mean of**  **control group** | **Mean of**  **patient group** |
| --- | --- | --- | --- | --- | --- | --- |
| S1-93 | 553 | TESNKKFLPFQQ | 564 | 41.1% | 11.8 | 724 |
| S1-111 | 661 | ECDIPIGAGICA | 672 | 49.70% | 22.1 | 963.9 |
| S1-113 | 673 | SYQTQTNSPRRA | 684 | 35.4% | 42.5 | 1191.9 |
| S2-19 | 794 | IKDFGGFNFSQI | 805 | 35.4% | 47 | 547.8 |
| S2-22 | 812 | PSKRSFIEDLLF | 823 | 45.0% | 44.6 | 2199.8 |
| S1-11 | 61 | NVTWFHAIHVSG | 72 | 0.0% | 3 | 11.2 |
| S2-7 | 722 | VTTEILPVSMTK | 733 | 0.0% | 4.3 | 2.8 |

**Supplementary Table 2. Result of BLAST analysis of S1-111 (organism: Mus).**

| **Description** | **Max Score** | **Total Score** | **Query Cover** | **E value** | **Per. ident** | **Acc. Len** | **Accession** |
| --- | --- | --- | --- | --- | --- | --- | --- |
| OCA2 protein, mutant type [Mus musculus castaneus] | 23.1 | 23.1 | 83% | 23 | 70 | 549 | BAQ59596.1 |
| carboxypeptidase A4 isoform X2 [Mus pahari] | 21.8 | 21.8 | 50% | 65 | 100 | 388 | XP_021046686.1 |
| carboxypeptidase A4 isoform X1 [Mus pahari] | 21.8 | 21.8 | 50% | 65 | 100 | 420 | XP_021046685.1 |
| carboxypeptidase A4 [Mus caroli] | 21.8 | 21.8 | 50% | 65 | 100 | 420 | XP_021020697.1 |
| carboxypeptidase A4 preproprotein [Mus musculus] | 21.8 | 21.8 | 50% | 65 | 100 | 420 | NP_082202.1 |
| Carboxypeptidase A4 [Mus musculus] | 21.8 | 21.8 | 50% | 65 | 100 | 420 | AAH61206.1 |
| carboxypeptidase A4 isoform X1 [Mus musculus] | 21.8 | 21.8 | 50% | 65 | 100 | 469 | XP_017177239.1 |
| fibrillin-2 [Mus musculus] | 21.8 | 35.2 | 100% | 65 | 61.54 | 1062 | AAA62177.1 |
| mKIAA4226 protein [Mus musculus] | 21.8 | 35.2 | 100% | 65 | 61.54 | 2225 | BAD90381.1 |
| fibrillin-2 isoform X2 [Mus musculus] | 21.8 | 35.2 | 100% | 65 | 61.54 | 2289 | XP_030106193.1 |


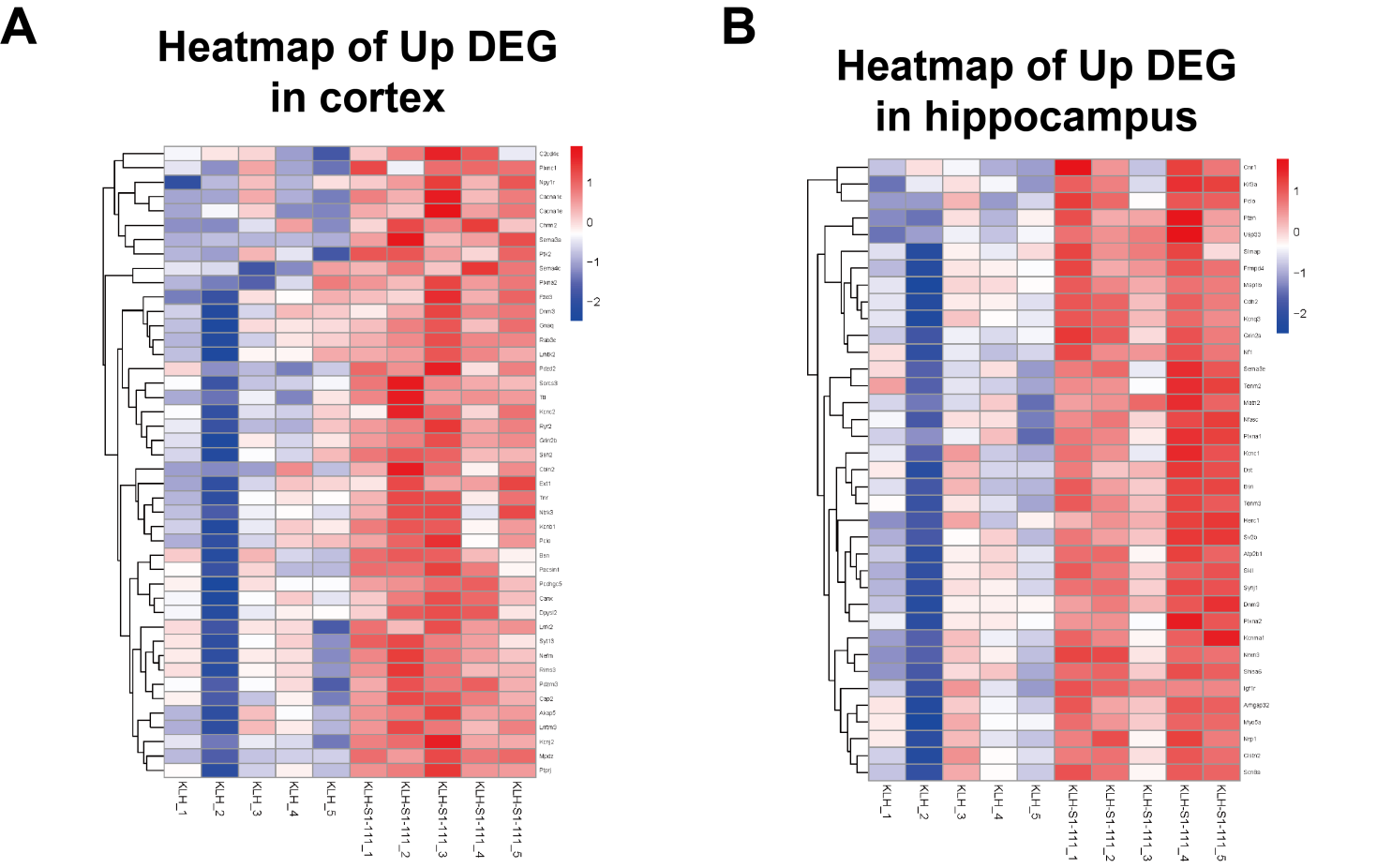


**Supplementary Figure 1.** **Heatmap of some neuron-related up-regulated genes in the cortex and hippocampus.**

**
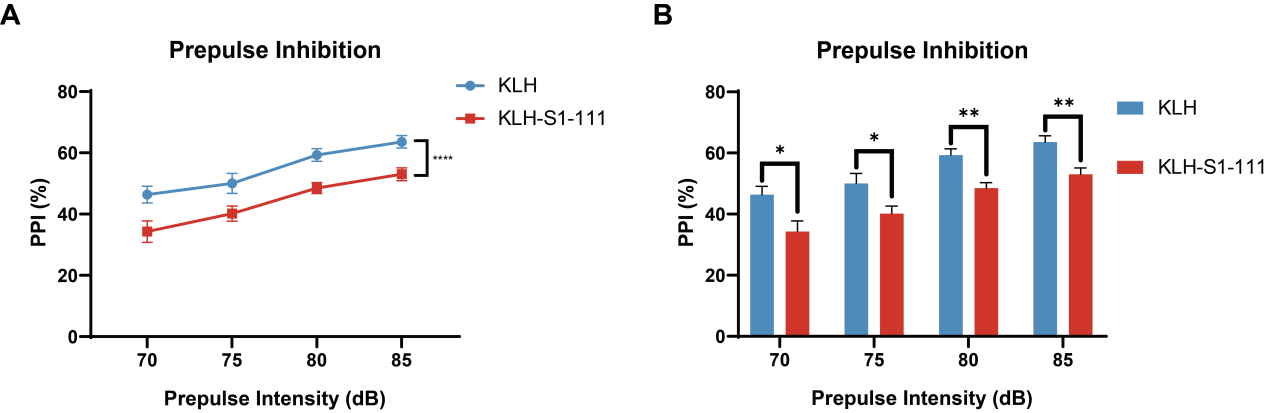
**

**Supplementary Figure 2. Behavioral effects of endogenous anti-KLH-S1-111 IgG antibodies in *ApoE*^-/-^ mice.**

**(A)** Line chart showing significantly decreased PPI in the K-S group compared to the KLH group, *p* < 0.0001, 2-way ANOVA with Bonferroni's test, and **(B)** *p* = 0.0165 at 70 dB, *p* = 0.0293 at 75 dB, *p* = 0.0015 at 80 dB, and *p* = 0.0029 at 85 dB, unpaired Student’s *t* test. **A-B.** N = 8/group, data are shown as the mean ± SEM.
